# Performance of P16^INK4a^ immunocytochemical stain in facilitating cytology interpretation of HSIL for HPV-positive women aged 50 and above

**DOI:** 10.3389/fonc.2024.1332172

**Published:** 2024-05-28

**Authors:** Jun Hou, Hui Du, Chun Wang, Fangbin Song, Xinfeng Qu, Ruifang Wu

**Affiliations:** ^1^ Department of Obstetrics and Gynecology, Peking University Shenzhen Hospital, Shenzhen, China; ^2^ Institute of Obstetrics and Gynecology, Shenzhen Peking University-Hongkong University of Science and Technology (PKU-HKUST) Medical Center, Shenzhen, China; ^3^ Shenzhen Key Laboratory on Technology for Early Diagnosis of Major Gynecologic Diseases, Peking University Shenzhen Hospital, Shenzhen, China; ^4^ Department of General Surgery, Shanghai General Hospital, School of Medicine, Shanghai Jiaotong University, Shanghai, China; ^5^ Department of Obstetrics and Gynecology, Sanming Project of Medicine in Peking University Shenzhen Hospital, Shenzhen, Guangdong, China

**Keywords:** P16 immunocytochemical stain, atrophy, cytology, high-grade squamous intraepithelial lesion (HSIL), menopause

## Abstract

**Background:**

Few articles have focused on the cytological misinterpretation of high-grade squamous intraepithelial lesion (HSIL). Due to estrogen deficiency, cervical epithelial cells in postmenopausal women tend to show atrophic change that looks like HSIL on Papanicolaou-stained cytology slides, resulting in a higher rate of cytological misinterpretation. P16^INK4a^ immunocytochemical staining (P16 cytology) can effectively differentiate diseased cells from normal atrophic ones with less dependence on cell morphology.

**Objective:**

To evaluate the role of P16 cytology in differentiating cytology HSIL from benign atrophy in women aged 50 years and above.

**Methods:**

Included in this analysis were women in a cervical cancer screening project conducted in central China who tested positive for high-risk human papillomavirus (hr-HPV) and returned back for triage with complete data of primary HPV testing, liquid-based cytology (LBC) analysis, P16 immuno-stained cytology interpretation, and pathology diagnosis. The included patients were grouped by age: ≥50 (1,127 cases) and <50 years (1,430 cases). The accuracy of LBC and P16 cytology in the detection of pathology ≥HSIL was compared between the two groups, and the role of P16 immuno-stain in differentiating benign cervical lesions from cytology ≥HSIL was further analyzed.

**Results:**

One hundred sixty-seven women (14.8%; 167/1,127) in the ≥50 group and 255 (17.8%, 255/1,430) in the <50 group were pathologically diagnosed as HSIL (Path-HSIL). LBC [≥Atypical Squamous Cell Of Undetermined Significance (ASCUS)] and P16 cytology (positive) respectively detected 63.9% (163/255) and 90.2% (230/255) of the Path-≥HSIL cases in the <50 group and 74.3% (124/167) and 93.4% (124/167) of the Path-≥HSIL cases in the ≥50 group. LBC matched with pathology in 105 (41.2%) of the 255 Path-≥HSIL cases in the <50 group and 93 (55.7%) of the 167 Path-≥HSIL cases in the ≥50 group. There were five in the <50 group and 14 in the ≥50 group that were Path-≤LSIL cases, which were interpreted by LBC as HSIL, but negative in P16 cytology.

**Conclusion:**

P16 cytology facilitates differentiation of Path-≤LSIL from LBC-≥HSIL for women 50 years of age and above. It can be used in the lower-resource areas, where qualified cytologists are insufficient, as the secondary screening test for women aged ≥50 to avoid unnecessary biopsies and misinterpretation of LBC primary or secondary screening.

## 1 Background

Diagnostics and treatment of cervical precancer for postmenopausal women are important to cervical cancer prevention in aged women because of the tendency of social aging in many countries. Cervical cytology remains the standard cervical cancer screening test worldwide for either primary or secondary screening. However, evidence shows that some atrophic changes in squamous and columnar epithelium may be misinterpreted as high-grade squamous intraepithelial lesion (HSIL) when analyzing the exfoliated cervical cells from postmenopausal women who are usually low in estrogen (1). However, many studies have evidenced that overexpression of P16 protein is positively related to transformative high-risk human papillomavirus (hr-HPV) infection and grade of cervical cell proliferation and can be an objective indicator for lesion grade. As a tumor suppressor that is highly related to HSIL and cervical cancer, P16 overexpression can be a biomarker for early diagnosis of squamous intraepithelial lesion (SIL) and evaluation of the lesion prognosis (2).

A P16 immunocytochemical stain technology was developed by Senying Biotechnology Co., Ltd. (Shenzhen, China), which uses P16^INK4a^ monoclonal antibodies (sy-a01) to stain exfoliated cervical cells that were diluted at a ratio of 1:4,000 on PathCIN^®^P16^INK4a^ automatic staining system (P16 immuno-stained cytology). This technology has been demonstrated to be more sensitive than and equally specific with liquid-based cytology (LBC) in the detection of grade II and above cervical intraepithelial neoplasia (CIN2+) (3). As it provides the cytopathologists with a more objective marker for cytology interpretation, it potentially reduces the subjective diversity in cytology interpretations from different cytologists and consequently reduces the reliance of cytology on cytologists’ experiences. This study aimed to demonstrate the performance of P16 immuno-stained cytology (or P16 cytology) in facilitating the differentiation of HSILs from atrophic lesions by comparing the concordance of P16-immuno-stained cytology with the LBC and histopathology diagnoses between the groups of women ≤50 and >50 years of age.

## 2 Materials and method

### 2.1 Study design, participants, and procedures

**Figure 1:**
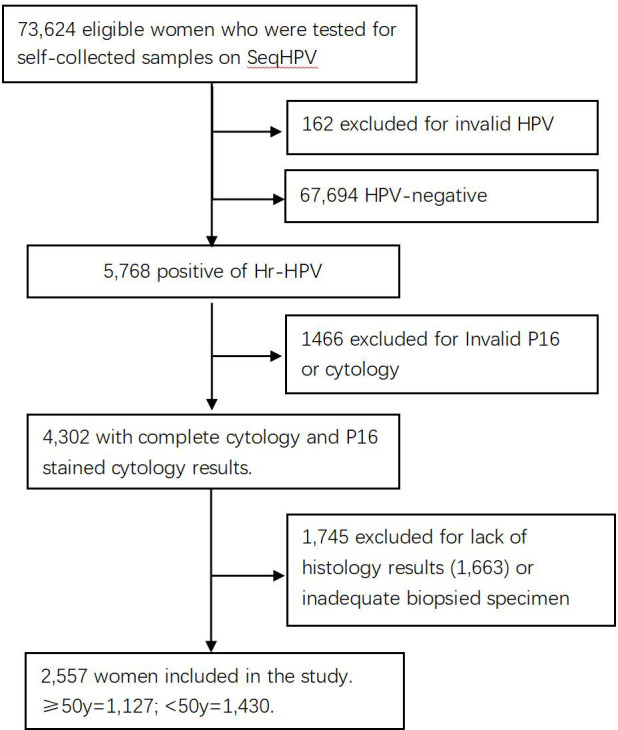
Flowchart of the study.

The subjects of the study were 73,624 women living in central China who were screened for cervical cancer by primary HPV testing in a population-based municipal cervical cancer screening program in November 2019. Those women were enrolled for screening because they were eligible: 30–64 years of age, not pregnant, without uterine or cervical resection, and consented to participate in the screening and this study by signing an electronic version of the informed consent form when they registered for participation on a website (www.curekeys.com). Eligible women were primarily tested for hr-HPV with SeqHPV assay on their self-collected samples. Women with HPV-negative results were advised to undergo regular screening by HPV assay after 3 years. Those positive for hr-HPV were recalled back for management following a protocol that required a cervical sample be collected first by the physician for LBC and P16 cytology analysis for all positive women, followed by multiple biopsies on women who were positive for HPV-16 and/or HPV-18, positive for the hr-HPV types other than HPV-16 and HPV-18 (other hr-HPV type) plus positive for acetic acid test, or positive for other hr-HPV types, and negative for acetic acid test but positive for LBC (≥ASCUS). Endocervical curettage (ECC) was performed on patients whose squamocolumnar junction zone (T-zone) could not be completely visible. Pathology analysis was conducted on the biopsies and ECC specimens. Included in this analysis were 2,557 women who had complete data on the primary HPV testing, LBC analysis, P16^INK4a^ immune-stained cytology (P16 cytology) interpretation, and pathology diagnosis. Women who were positive for other hr-HPV types but normal for both LBC and P16 cytology, or positive for any type but did not have results of LBC, P16 cytology, or histopathology, mainly due to sample reasons, were excluded from this study (**Figure 1**). The study was approved by the Institutional Review Board (IRB) of BGI Institute and the Ethics Committee of Peking University Shenzhen Hospital (PUSH, No. 2018035).

### 2.2 Sampling and HPV testing

After successful registration, which confirmed eligibility for participation in the primary screening, women were screened in the sampling sites temporarily set up in the communities according to the number of registered women in the relevant communities or nearby medical facilities (the screening sites). At the screening site, eligible women were guided to collect cervical/vaginal samples for themselves in sampling rooms by referring to the graphic self-sampling instruction with texts. A conical-shaped brush was used for self-sampling. If any woman had a problem with self-sampling, an on-site medical provider would give personal instruction. The collected sample was applied on an FTA-Illusive-card (GE) for HPV testing on SeqHPV (BGI-Shenzhen) by a reference laboratory of BGI-Shenzhen. SeqHPV is a next-generation sequencing (NGS)-based HPV testing assay that uses multi-plex PRC to amplify DNA and NGS for HPV genotyping (3). This assay can detect and report 14 hr-HPV genotypes, including HPV-16, HPV-18, HPV-31, HPV-33, HPV-35, HPV-39, HPV-45, HPV-51, HPV-52, HPV-56, HPV-58, HPV-59, HPV-66, and HPV-68. SeqHPV had been validated to be equally sensitive and specific with Cobas4800 when tested on either provider-collected or self-collected samples (4) and to work well with FTA cards (a hard sample processing card). It has been licensed by China Food and Drug Administration (CFDA) for clinical use.

### 2.3 LBC and P16^INK4a^ immuno-stained cytology

LBC and the P16^INK4a^ immuno-stained cytology (Senying Biotechnology Co., Ltd., Shenzhen, China) were used for research purposes and as the secondary screening in the triage of the women who were positive for 12 other types of hr-HPV plus negative for acetic acid test. The cervical sample was collected by the provider and then put into a vial containing cell preservation liquid provided by Senying. The samples were shipped to the Senying laboratory for processing: part of each sample was processed with P16^INK4a^ immunocytochemical stain, and the remaining sample was processed for standard Papanicolaou stains. Both the P16^INK4a^ immuno- and Papanicolaou-stained cytology slides were reviewed and interpreted by two senior cytopathologists who were blinded to each other’s interpretation.

Following The Bethesda System (TBS) classification standards (5), LBC interpretations were reported as negative for squamous intraepithelial lesion (NILM), atypical squamous cells of undetermined significance (ASCUS), atypical glandular cells (AGC), low-grade squamous intraepithelial lesion (LSIL), atypical squamous cells-cannot exclude HSIL (ASC-H), HSIL, or squamous cell carcinoma (SCC) accordingly. P16 cytology positive result was reported when at least one cell was found to have P16^INK4a^ immuno-stained substance in the nucleus or cytoplasm. Quality control was conducted after the two cytopathologists completed their review of the slides, on which all cases with inconsistent interpretations by the cytologists were selected to resolve consistent interpretations via discussions between the two cytopathologists.

### 2.4 Colpo/biopsy and histopathology diagnostics

For women who needed biopsies according to the protocol for positive management, multiple biopsies were obtained at the site with colposcopically suspected lesions and the opposite sites, or randomly at the squamocolumnar junction zone in four quadrants of the cervix if no lesion was suspected. ECC was performed on patients whose squamocolumnar junction zone could not be completely visible under colposcopy (6).

All the pathology slides were analyzed by a senior pathologist from PUSH who performed pathology analysis for several international clinical trials. Pathology slides were analyzed while blinded to the results from both the P16 cytology and LBC tests. Histological diagnoses for cervical lesions were reported following a two-grade classification system, according to which the cervical lesions were classified as LSIL and HSIL. This system was adopted because many studies demonstrated that different grades of CIN are not the different stages of a cervical lesion development but the two obviously distinguishable pathological processes, and the two-grade classification matches with the bio-behavior of HPV that causes pathology changes in human cells and is with better duplicability (7–9).

### 2.5 Statistics

Results from LBC and pathology were compared to demonstrate the bias of LBC on the interpretation of HSILs in women ≥50 years of age, and P16 cytology results of the cases with LBC-LSIL and LBC-HSIL were analyzed using the relevant pathology diagnosis as the endpoint (Path-LSIL and Path-HSIL). SPSS 26.0 statistical software was used for data analysis. The chi-square test was used to compare the differences in various rates. Differences in sensitivity and specificity along with 95% CI were calculated using McNemar’s test, and a p-value of <0.05 was considered statistically significant for all analyses.

## 3 Results

Among the 73,624 primarily screened women, 73,462 had valid results for HPV primary testing after excluding 162 for failure of HPV testing. Of the 73,462, 5,768 were positive for primary HPV testing, and 2,557 of those positive results had complete data of HPV, LBC, P16 cytology, and histopathology results and were included in the analysis for the purpose of this study (the analytic cases). Patients who were primarily positive for 12 other types of hr-HPV but normal in cytology and P16 cytology, or abnormal in the two cytology tests but did not return for colposcopy, were excluded from this analysis.

Of the 2,557 analytic cases, 1,430 were younger than 50 years and included in the <50 group, while the remaining 1,127 were aged 50 and above and included in the **≥**50 group. HSIL was pathologically confirmed (Path-HSIL) on 255 and 167 positive women in the <50 group and ≥50 group, respectively. When analyzed in age groups with LBC**≥**ASCUS and P16 cytology positive results as the cutoff, LBC and P16 cytology respectively detected 63.9% (163) and 90.2% (230) of the 255 Path-HSIL cases in the <50 group, while in the **≥**50 group, LBC and P16 cytology detected 74.3% (124) and 93.4% (156) of the 167 Path-HSIL cases, respectively (**Table 1**).

**Table 1:** The detection rate of LBC≥ASCUS and P16+ in two groups for detection of Path-HSIL.

Groups	≥50	<50
	Path-≥HSIL	Path-≥HSIL
≥ASCUS	74.3 (124/167)	63.9 (163/255)
P16+	93.4 (156/167)	90.2 (230/255)
P	0	0

LBC, liquid-based cytology; HSIL, high-grade squamous intraepithelial lesion.

When looking at the number of HSIL cases reported by LBC and pathology, we observed that HSIL from LBC (LBC-HSIL) and pathology (Path-HSIL) in the <50 group were 110 and 255 cases, respectively (**Table 2A**), while LBC- and Path-HSIL in the ≥50 group were 107 and 167 cases, respectively (**Table 2B**). However, when looking at the concordance of LBC and pathology detecting HSIL, we found that LBC matched with pathology in 41.2% (105/255) of the HSIL cases from the <50 age group and 55.7% (93/167) of such cases from the ≥50 group.

**Table 2A:** LBC-LSIL/HSIL vs. Path-LSIL/HSIL in ≥50 group, n (%).

	LBC-≤LSIL	LBC-≥HSIL	Total
Path-≤LSIL	946 (98.5)	14 (1.5)	960 (100)
Path-≥HSIL	74 (44.3)	93 (55.7)	167 (100)
Total	1,020 (90.5)	107 (9.5)	1,127 (100)

**Table 2B:** LBC-LSIL/HSIL vs. Path-LSIL/HSIL in <50 group, n (%).

	LBC-≤LSIL	LBC-≥HSIL	Total
Path-≤LSIL	1,170 (99.6)	5 (0.4)	1,175 (100)
Path-≥HSIL	150 (58.8)	105 (41.2)	255 (100)
Total	1,320 (92.3)	110 (7.7)	1,430 (100)

Comparison of **Table 2A** and **Table 2B**: X^2 ^= 2.635, p = 0.105 for LBC and X^2^ = 4.155, p = 0.042 for pathology.

LBC, liquid-based cytology; LSIL, low-grade squamous intraepithelial lesion; HSIL, high-grade squamous intraepithelial lesion.

What interested us were the five (0.4%) and 14 (1.5%) Path-≤LSIL cases in the <50 and ≥50 groups, respectively, that were reported by LBC as HSIL cases (LBC-HSIL/Path-≤LSIL cases), with significant difference between the two groups (p = 0.042). Further analysis showed that all the five LBC-HSIL/Path-≤LSIL cases in the <50 group and 14 such cases in the ≥50 group were negative for P16 cytology, of which the five from the <50 group and 13 from the ≥50 group were pathologically confirmed as normal, and one from the ≥50 group was Path-LSIL (**Table 3**). These results indicate that P16 cytology is contributive in differentiating benign lesions from HSIL for cytology (LBC) on women ≥50 years of age.

**Table 3:** P16 cytology performance in detection LBC-HSIL+ cases in the two age groups.

Pathology		Normal		LSIL		HSIL+	
	P16 cytology	P16+	P16−	P16+	P16−	P16+	P16−
LBC-HSIL+ ≥50 (n = 107)		0	**13**	0	**1**	90	3^*^
LBC-HSIL+ <50 (n = 110)		0	**5**	0	0	104	1^*^

LBC, liquid-based cytology; HSIL, high-grade squamous intraepithelial lesion; LSIL, low-grade squamous intraepithelial lesion.

^*^The pathological results of the above four cases were CIN2 and Path-P16 negative. The bold values means the cases which LBC-≥HSIL/Path-≤LSIL but P16-.

**Table 3** also shows that there were one and three LBC-HSIL/Path-HSIL cases in the <50 and ≥50 groups, respectively, that were negative for P16 cytology. All those cases were pathologically graded as CIN2.

## 4 Discussion

Our prior study has demonstrated that P16 cytology is better than LBC in the sensitivity and specificity for the detection of CIN2+ (10) but less dependent on cell morphology, which makes it more applicable in lower-resource areas where experienced and acknowledgeable cytologists are insufficient. In this study, we found that P16 cytology is advantageous in facilitating cytologists to differentiate benign lesions from LBC-≥HSIL in women ≥50 years of age. Due to obvious decreased levels of estrogen in women during perimenopause, some atrophic cervical cells are usually included in the cervical samples for cytology, resulting in its potential misinterpretation as HSIL (11). As the atrophic cells have smaller portions of cytoplasm, it is easy to be confused with HSIL cells on Papanicolaou-stained cytology slides, and this has always been challenging to cytologists, especially to the inexperienced ones in lower-resource regions.

Our analysis shows that the rate of LBC-reported false HSILs is significantly higher in the ≥50 group than in the <50 group. This result is consistent with many studies that reported that among the cases that returned for colposcopy/biopsies for LBC-≥HSIL, the average age of the cases’ normal pathology was higher than those pathologically diagnosed as HSIL (12–14). In a study on LBC-ASC-H cases (15), Halford and coauthors reported that the rates of CIN2 were 55.8% and 37.5% among patients aged <50 and ≥50 years, respectively, with significant differences. Other studies attributed the higher rate of inconsistency between LBC-HSIL and Path-Normal among women aged ≥50 years to cervical atrophic changes caused by the drop in estrogen levels, which often led to many parabasal cells and basal cells being stained dark on cytology views (16–18). LBC may possibly misinterpret cervical atrophic cells as HSIL or even cancer (19). The atrophy changes on squamous epithelium make the Papanicolaou test less precise and specific in the detection of HSIL (20, 21). Recent studies found that P16/Ki67 double-stained cytology performed high profiles for CIN2+ in postmenopausal women cytologically reported with ASCUS (22). Since misinterpretation of atrophic cells as HSIL+ would not only bring heavy psychological pressure to women but also lead to unnecessary biopsies, the performance of P16 immunocytochemical stain in the facilitation of the interpretation of cytology for women aged 50 or above is worth addressing for its clinical application.

**Figure 2:**
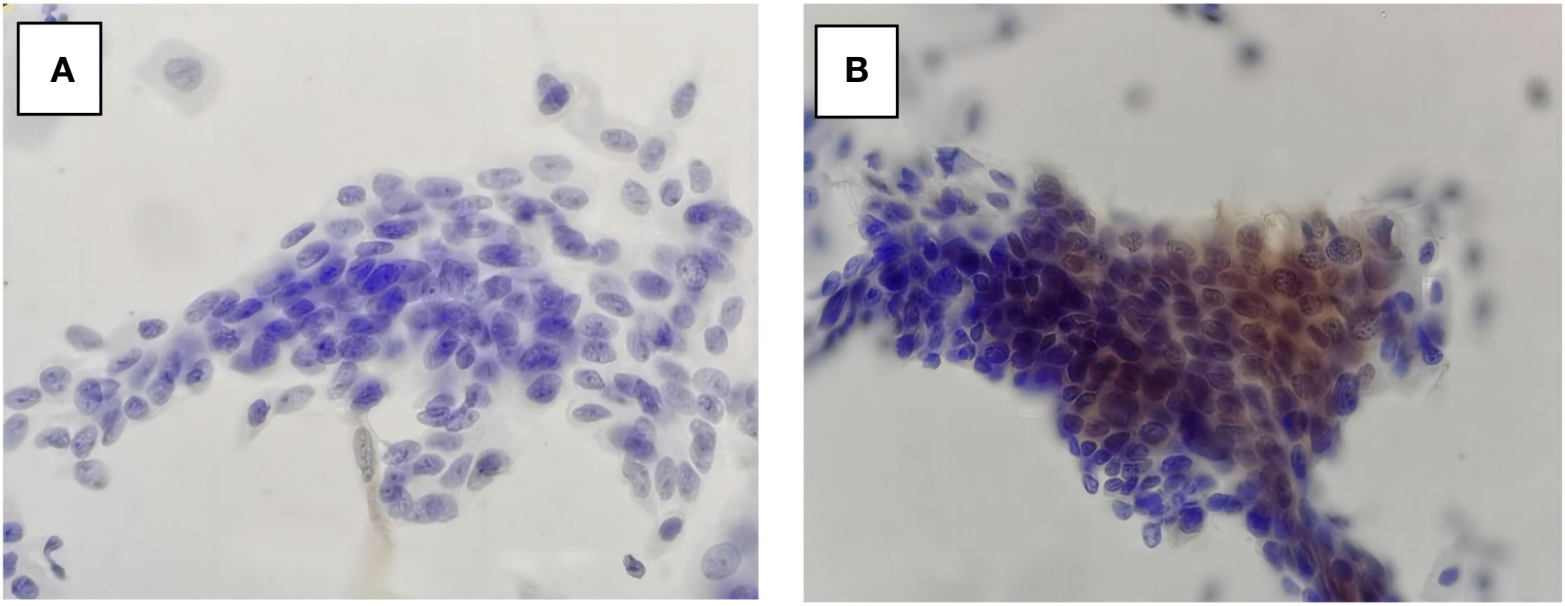
P16 cytology in LBC-≥HSIL/Path-≤LSIL **(A)** and LBC-≥HSIL/Path-≥HSIL **(B)**. LBC, liquid-based cytology; HSIL, high-grade squamous intraepithelial lesion; LSIL, low-grade squamous intraepithelial lesion.

In our study, P16^INK4a^-stained cytology (P16 cytology) performs well in differentiating Path-≤LSIL from LBC-≥HSIL (**Figure 2**). Those findings are important for further studies and clinic services since P16 cytology helps avoid the atrophic changes of the cervical cells from aged women being misinterpreted as ≥HSIL by LBC. P16-immuno-stain also contributes to the indication of the invisible HSIL+ under colposcopy (23). In our study, all the LBC-≥HSIL cases that were also positive for P16 were pathologically diagnosed as ≥HSIL. Further analysis of the four Path-≥HSIL cases that were negative for P16 showed that all of them were pathologically graded as CIN2. We do not have data to confirm whether P16 negative results in those cases are potentially caused by hypermethylation (24, 25) or a regressive tendency of those CIN2 cases. The persistence of HPV infections also should be given great importance, as it is related to the persistence and recurrence of HSIL (26, 27). Further study is needed to answer those questions. The findings in our study suggest that P16 immuno-stain can play an important role in avoiding either overdiagnosis or misdiagnosis. For many years, investigators have endorsed finding proper technology for secondary screening that can keep enough sensitivity for the detection of HSIL but avoid unnecessary biopsies.

Our previous studies demonstrated that the detection rate of P16 cytology is as same as that of HPV testing and LBC analysis for Path-HSIL and above and can be used as the secondary screening test for positive women after primary HPV screening (28). Those studies also indicated that P16 cytology as well as cytology can find abnormal cells that may potentially progress to carcinomas and is better than HPV testing in indicating precancer (29). P16 cytology can be tested at the same time with LBC on the same sample and is advantageous as the secondary screening after primary hr-HPV testing in improving the accuracy of cytology analysis (30). Our analysis in this study further demonstrated the important advantage of P16 cytology in facilitating cytologists to differentiate atrophic changes from HSIL.

In conclusion, our study demonstrated that P16 cytology facilitates differentiation of Path-≤LSIL from LBC-≥HSIL for women 50 years of age and above. It can be used in the lower-resource areas where qualified cytologists are insufficient as the secondary screening test for women aged ≥50 to avoid unnecessary biopsies and misinterpretation of LBC primary or secondary screening.

This study is one of the few retrospective studies on triage in women with LBC-≥HSIL with P16^INK4a^ immunocytochemical stain. It contributes a basis for further studies in the relevant area. However, our study has limitations: patients were grouped by age rather than by menstruation status; thus, there is a lack of proof for the histological atrophy changes. It could be more evident if the LBC and P16 cytology results were from primary screening.

## Data availability statement

The raw data supporting the conclusions of this article are available from the corresponding author on reasonable request.

## Ethics statement

The studies involving humans were approved by The Ethics Committee from Peking University Shenzhen Hospital (No.2018035). The studies were conducted in accordance with the local legislation and institutional requirements. The participants provided their written informed consent to participate in this study.

## Author contributions

JH: Data curation, Formal analysis, Writing – original draft. HD: Writing – review & editing. CW: Data curation, Methodology, Writing – original draft, Resources, Supervision. FS: Investigation, Methodology, Resources, Supervision, Writing – review & editing. XQ: Conceptualization, Data curation, Formal analysis, Investigation, Methodology, Writing – review & editing. RW: Data curation, Formal analysis, Funding acquisition, Methodology, Project administration, Resources, Supervision, Writing – original draft, Writing – review & editing.
